# A new database of chestnut DNA fingerprints for genetic diversity assessment, precise varietal identification, and traceability

**DOI:** 10.1093/database/baaf056

**Published:** 2025-09-24

**Authors:** Ivan Fruggiero, Alessandro Maisto, Sara Passaro, Domenico Gentile, Angelina Nunziata, Nunzio D’Agostino

**Affiliations:** Department of Agricultural Sciences, University of Naples Federico II, piazza Carlo di Borbone 1, 80055 Portici, Napoli, Italy; Interuniversity Center for Studies on Bioinspired Agro-Environmental Technology, University of Naples Federico II, piazza Carlo di Borbone 1, 80055 Portici, Napoli, Italy; Department of Agricultural Sciences, University of Naples Federico II, piazza Carlo di Borbone 1, 80055 Portici, Napoli, Italy; Interuniversity Center for Studies on Bioinspired Agro-Environmental Technology, University of Naples Federico II, piazza Carlo di Borbone 1, 80055 Portici, Napoli, Italy; Council for Agricultural Research and Economics (CREA), Research Center for Olive, Fruits, and Citrus Crops, 81100 Caserta, Italy; Council for Agricultural Research and Economics (CREA), Research Center for Olive, Fruits, and Citrus Crops, 81100 Caserta, Italy; Council for Agricultural Research and Economics (CREA), Research Center for Olive, Fruits, and Citrus Crops, 81100 Caserta, Italy; Department of Agricultural Sciences, University of Naples Federico II, piazza Carlo di Borbone 1, 80055 Portici, Napoli, Italy; Interuniversity Center for Studies on Bioinspired Agro-Environmental Technology, University of Naples Federico II, piazza Carlo di Borbone 1, 80055 Portici, Napoli, Italy

## Abstract

The European chestnut (*Castanea sativa* Mill., Fagaceae) is ecologically and economically important, particularly in countries like Italy, Greece, Spain, and Turkey, where it supports rural economies and ecosystems. Accurate varietal recognition is crucial for managing chestnut groves but is hindered by the limitations of traditional methods, which require costly expertise and struggle to identify young, dormant, or scion trees. Recent advances in molecular tools, particularly single nucleotide polymorphism (SNP) markers identified through Kompetitive Allele-Specific PCR (KASP) technology, have transformed cultivar identification. To harness this potential, we developed KASTRACKdb, a genetic fingerprinting database for European chestnut that now integrates genotypic and phenotypic data for 150 chestnut accessions. Designed to translate KASP analysis results into practical and actionable insights, KASTRACKdb serves as a powerful tool for cultivar identification and management. The database offers three primary query modes and is designed for continuous upgrades, serving a crucial role in cataloguing the genetic diversity of chestnut trees, characterized by broad geographic distributions and significant genetic variation. This diversity is critical for conservation and breeding programs, enabling precise varietal identification and traceability to protect intellectual property, verify authenticity, and support the commercialization of high-value cultivars.

**Database URL**: KASTRACKdb is available online at https://kastrack.crea.gov.it/kastrackdb/?lang=en.

## Introduction

The European chestnut (*Castanea sativa* Mill.) is a species of significant ecological and economic value, thriving across hilly and mountainous regions of Europe and Asia Minor. Historically, it has been vital to rural economies, offering durable timber and nutritious fruits, while more recently gaining attention for its nutraceutical benefits [1,2].

Chestnut production in Europe has experienced a steady decline over recent decades, largely driven by increasing industrialization and its socio-economic consequences. The economic boom following the Second World War triggered widespread rural depopulation, as people migrated towards urban industrial centres in search of better opportunities. This demographic shift, alongside evolving lifestyles and dietary habits significantly impacted both the cultivation and commercialization of chestnuts. At the same time, traditional uses of chestnut and its by-products, such as timber for poles and telegraph posts, as well as tannin extraction, were increasingly replaced by alternative materials and modern technologies. These trends were further exacerbated by the emergence of destructive pathogens and pests, most notably chestnut blight (*Cryphonectria parasitica*) and the invasive chestnut gall wasp (*Dryocosmus kuriphilus* Yasumatsu), both of which severely compromised chestnut cultivation [3,4].

As a result, many fruit-producing chestnut groves were gradually abandoned, converted into coppice stands, or replaced entirely with fast-growing tree species. In Italy, for instance, only 38% of chestnut groves were managed as coppices in 1950. By 2008, before the widespread impact of *D. kuriphilus*, this figure had surged to 81%, indicating a marked transformation in forest management practices [5].

Chestnut cultivars are primarily propagated through grafting, a fundamental practice likely introduced to Europe before the 15th century [6]. This method has resulted in a strong correlation between cultivar identity and genotype, which simplifies the genetic characterization of traditional cultivars. More recently, *in vitro* micropropagation of scions has gained prominence, as it ensures the production of more uniform and disease-resistant plants. This advancement also highlights the need and opportunity for precise genotyping of scions [7,8].

Italy serves as a vital reservoir of chestnut genetic diversity, hosting numerous local varieties that have been preserved over generations through the dedicated efforts of local growers. Traditional chestnut varieties were typically named based on their geographic origin, ripening period, or intended use. This naming practice often led to confusion, resulting in cases of homonymy (different genetic variants sharing the same name) and synonymy (the same genetic variant known by multiple names) [9] However, this rich yet complex genetic heritage is increasingly at risk due to biodiversity loss driven by both natural and anthropogenic factors. This pressing challenge underscores the urgency for the characterization and conservation of local chestnut varieties [1,10]. Effective conservation efforts require a combination of *ex situ* and *in situ* strategies. For chestnut trees, *ex situ* conservation primarily involves establishing and maintaining live collections in field gene banks, ensuring the preservation of genetic diversity [11]. *In situ* methods involve supporting local farmers in cultivating traditional chestnut varieties and protecting wild relatives in their natural habitats [12]. Collaboration with local communities is vital, as farmers and indigenous groups play a critical role in preserving traditional knowledge and practices related to chestnut diversity [13]. By integrating these elements, conservation strategies can secure the preservation and sustainable use of chestnut diversity for future generations.

Implementing effective conservation strategies begins with identifying and characterizing genetic diversity. Traditionally, chestnut varieties have been distinguished by morphological traits; however, this approach often lacks the accuracy required for reliable identification [14].

In recent years, molecular markers such as random amplified polymorphic DNA and microsatellites (simple sequence repeats) have been widely used for genetic characterization of chestnut [15,16]. The availability of the chestnut genome [17–20] provides a valuable reference that enhances genotyping efforts, enabling the development of high-resolution genetic markers, such as single nucleotide polymorphisms (SNPs). These markers are essential for assessing genetic diversity, tracing parentage, and conducting more precise varietal identification.

Kompetitive Allele-Specific PCR (KASP) technology has gained prominence as an efficient and cost-effective tool for SNP-based genotyping [21–23]. By discriminating specific alleles through a competitive polymerase chain reaction (PCR) process, KASP offers a streamlined, fully scalable workflow. Combining the accuracy of fluorescence-based methods with reduced costs, KASP is particularly well-suited for varietal identification in species like chestnut [1], providing both reliability and accessibility for large-scale applications.

There is a clear need for a tool that could quickly and effectively translate KASP analysis results into actionable insights. In response to this demand, we developed a comprehensive database, named KASTRACKdb, for the genetic fingerprinting of European chestnut, enabling efficient analysis and interpretation of genetic data. A genetic fingerprinting database is vital for several key reasons. It plays an essential role in assessing the genetic diversity of chestnut trees, which are characterized by broad geographic distributions and long lifespans, resulting in considerable genetic variation. Cataloguing this diversity is essential for both conservation and breeding programs. The genetic data within the database supports these efforts by facilitating the identification of individuals carrying desirable allelic combinations associated with beneficial traits and guiding genetic improvement initiatives. It also helps conserve genetic resources by pinpointing and preserving unique or endangered varieties, both through *ex situ* and *in situ* conservation strategies. The database also ensures precise varietal identification and traceability, which are crucial for protecting intellectual property, verifying authenticity, and supporting the commercialization of high-value cultivars. Thanks to the availability of genetic information, the chestnut nursery sector can enhance its sustainable growth through stringent controls over propagation materials, thereby bolstering the reputation and reliability of nurseries among consumers and stakeholders. Additionally, providing comprehensive and trustworthy information about the varietal heritage of chestnut trees will facilitate informed transactions in the buying and selling of chestnut groves. Together, these measures will promote a more dynamic and transparent market, driving innovation and long-term development in the sector.

## Materials and methods

### Data collection and annotation

**Figure 1.**
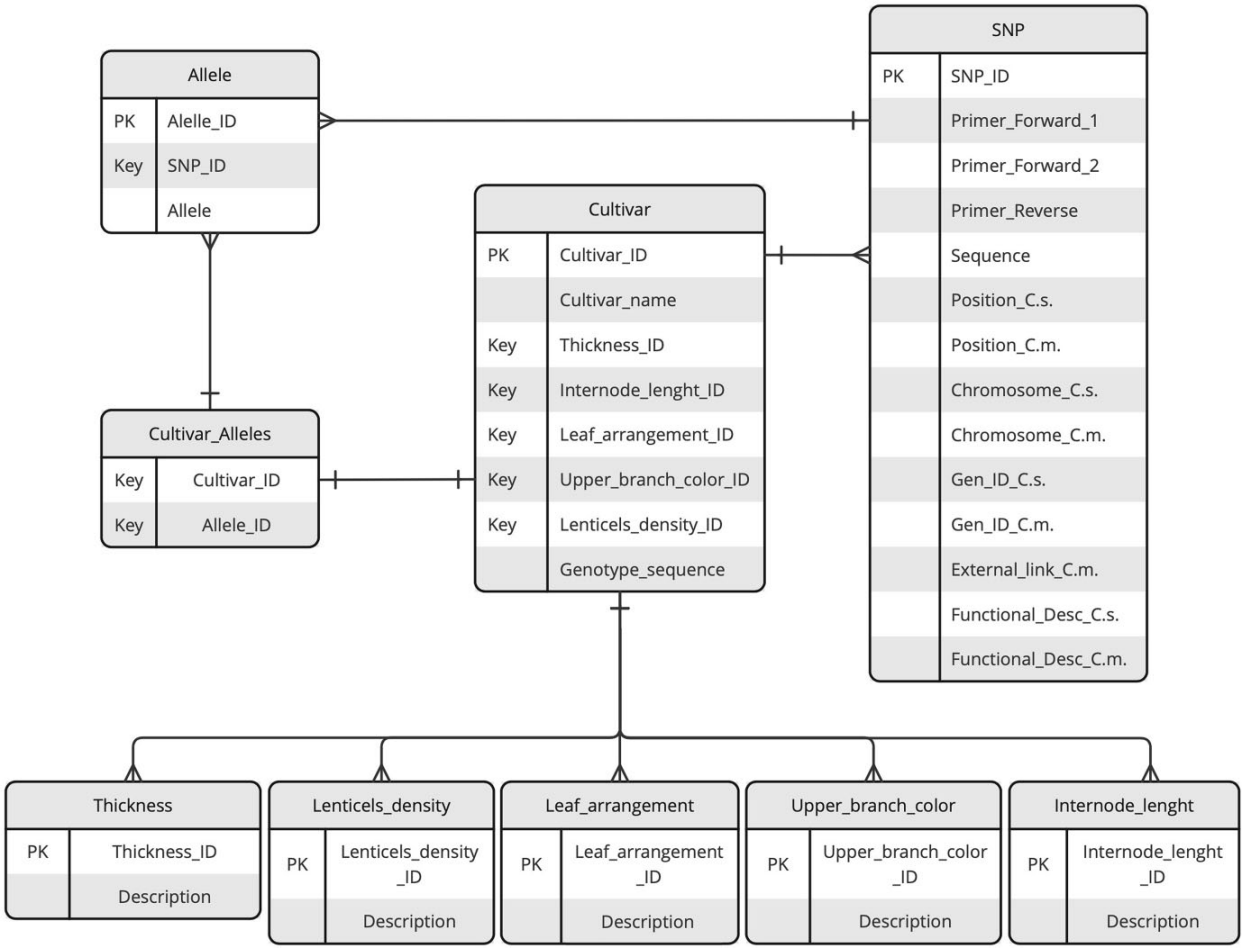
ER diagram of the KASTRACK database. The diagram illustrates key entities, including SNPs, cultivars, alleles, and additional tables central to organizing phenotypic data. Relationships between entities are represented by lines with connectors, highlighting the data interconnections and dependencies.

The structure and organization of the data repository are illustrated through the Entity–Relationship (ER) diagram provided in Fig. 1. This diagram serves as a visual representation of the repository design, detailing the key entities, their attributes, and the relationships between them.

KASP genotyping data, along with the corresponding sequence primers for the KASP assay and SNP-flanking sequences, were imported into the database in comma-separated values format. Additionally, phenotypic data for five morphological traits—branch thickness, internode length, leaf arrangement, upper branch colour, and lenticels density—were included. These traits were described and standardized following the guidelines established by the International Union for the Protection of New Varieties of Plants, ensuring consistency and accuracy in data representation. Data on selected SNPs and KASP assay design were obtained from a previous study by Nunziata *et al*. [1]. The genotyping of the 150 individuals included in the database was conducted following the methods previously described [1].

The sequences containing the SNPs were aligned to the *C. sativa* and *C. mollissima* reference genomes [17,18], integrating detailed information such as chromosome position, gene ID, and functional description, with gene details included if the SNP falls within a gene. For each SNP marker, the database also provides information on the forward and common primers used in KASP assays.

### Database architecture

KASTRACKdb has been built on an Ubuntu (v24.04)-based machine’s Apache HTTP Server (v2.4.54). MySQL 8.0 is used as the database management system to store and manage the data. The front-end interface is designed to provide access to the data contained in the database through a user-friendly graphical interface. This interface is based on a combination of programming languages to ensure both functionality and aesthetic appeal. Hypertext Preprocessor (PHP) connects the interface to the server, dynamically generating content. HTML structures web pages for clear navigation, while CSS enhances visual design and layout. JavaScript adds interactivity and responsiveness, creating an engaging and efficient user experience. By leveraging standard web technologies, the platform remains accessible across various devices and requires no specialized software, ensuring ease of use for all users. The online version of KASTRACKdb is accessible at https://kastrack.crea.gov.it/kastrackdb/?lang=en and requires no authentication or registration. All data within KASTRACKdb are freely available online without any restrictions, ensuring open access for all users.

### Integration of bioinformatics tool

KASTRACKdb integrates several bioinformatic tools to enhance its analytical capabilities. MAFFT (v7.525 https://mafft.cbrc.jp/alignment/software/) is utilized both to perform multiple alignments of nucleotide sequences, ensuring accurate and efficient sequence comparison, and to generate a guide tree in Newick format via the –treeout option. The resulting tree is then rendered in SVG format using the Python toolkit ETE3 (v3.1.2). These integrated resources enable advanced analyses, such as generating a tree diagram that illustrates the relationships among chestnut accessions.

### Database content and use

#### Search functions

The database includes genotypic and phenotypic data points for 150 chestnut accessions, including 38 SNPs with comprehensive information on KASP primers, sequences, and positions. Designed to support a wide range of genotype analysis tasks, it offers three distinct query modes, each tailored to address the diverse practical scenarios researchers may encounter throughout the genotyping process. Users can initiate analyses using various input types, including genetic data, morphological traits, FASTA sequences, or even cultivar names. This versatility enables targeted interrogations, ensuring that the results are directly aligned with the specific starting point and research objectives.

#### Combined search

**Figure 2.**
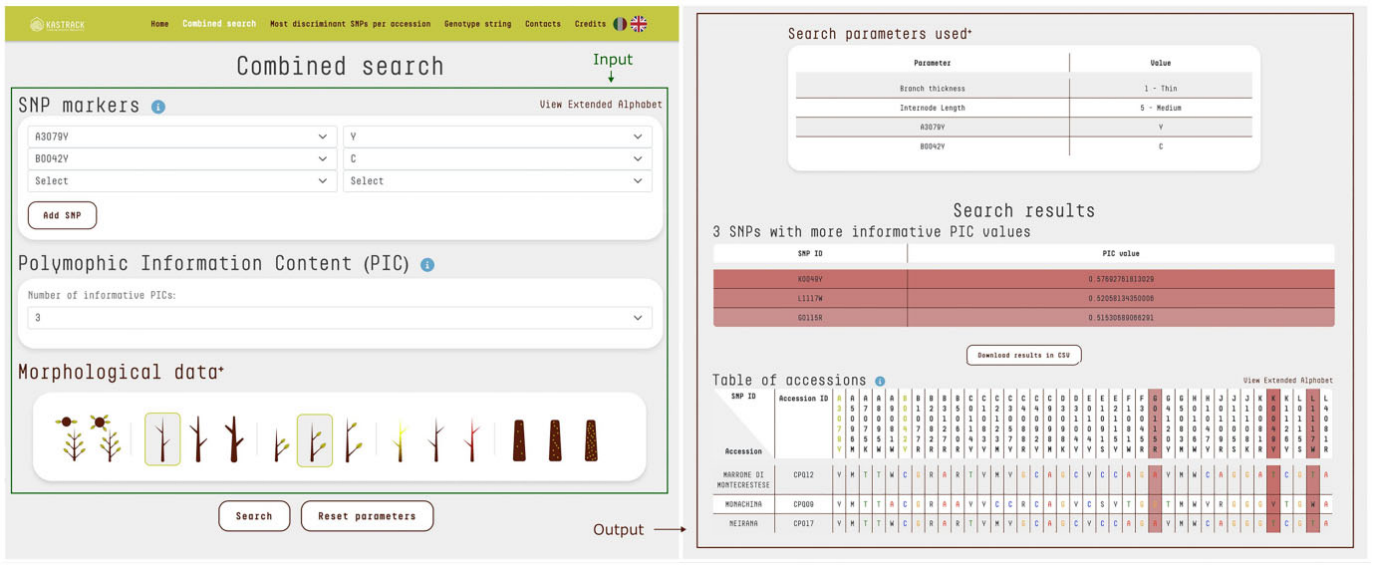
The combined search input consists of SNP markers, the selected number of SNPs with the highest PIC, and morphological trait data. The query was as follows: SNP marker A3079Y with allele Y and SNP marker B0042Y with allele C. The number of SNPs with the highest PIC values was set to 3 by default. Morphological data included branch thickness: thin and internode length: medium. The output displays a table of accessions (*N* = 3) matching the query criteria. SNP loci specified in the input are highlighted with a different text color, while SNPs with the highest PIC values are evidenced by shades. Their respective PIC scores are also reported. For clarity, each nucleotide is displayed in a distinct colour, while letters from the extended alphabet, representing heterozygosity, are uniformly styled for consistency.

The database primary feature enables users to access KASP data and phenotypic data points either individually or in combination, facilitating the efficient retrieval of accessions that meet specific criteria. The user-friendly search interface accepts various inputs, including one or more SNP markers with their respective alleles, the desired number of markers with the highest polymorphic information content (PIC) values [24] to display in the results, and the morphological traits observed in the sample being analysed. This functionality enhances precision and flexibility in data retrieval. The output is presented in a table that lists the accessions filtered according to the user-defined parameters, along with their annotated alleles for each marker in the database (Fig. 2). Furthermore, the system generates a list of SNPs with the highest PIC values, providing essential insights to help operators design more effective and targeted KASP analyses.

### Most discriminant SNPs per accession

**Figure 3.**
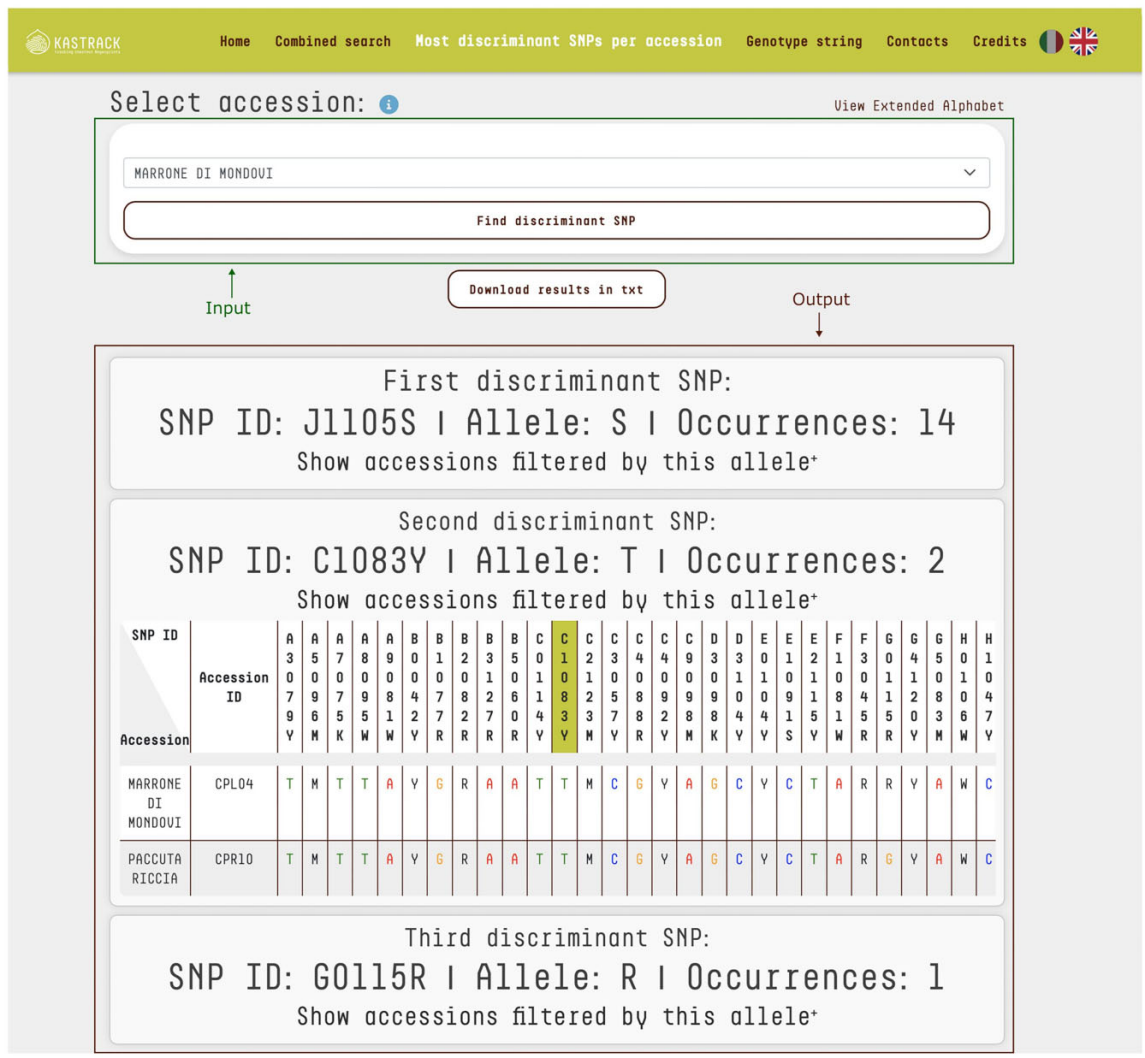
‘Most Discriminant SNPs per Accession’ query mode. The top section features a drop-down menu enabling users to select a specific accession for identification. The bottom section displays the output as tables listing the SNPs necessary to uniquely identify the selected accession. For each SNP, the table provides its ID, corresponding allele, and the number of occurrences across the database. For the input accession ‘Marrone di Mondovi’, marker J1105S with allele S distinguishes 14 accessions, with the full list accessible via the ‘Show accessions filtered by this allele’ link. The second marker, C1083Y with allele T, further reduces the dataset to just 2 accessions. Finally, marker G0115R with allele R is unique to ‘Marrone di Mondovi’. Together, these three markers uniquely identify ‘Marrone di Mondovi’ among the 150 accessions in the database. The system uses an iterative algorithm that progressively refines the list of candidate accessions, highlighting the most informative SNPs for precise discrimination.

An additional feature on the platform is the ability to identify the most discriminating SNPs for each accession. By selecting an accession from the database, users can pinpoint, which SNPs most effectively distinguish it. The algorithm uses an iterative filtering approach to analyse the chosen accession’s genotype, identifying the marker–allele combination that appears the least frequently across the database. The list of potential accessions is then narrowed down based on that SNP, with the search process being repeated on the filtered subset until the accession can be uniquely identified. This method ensures an efficient and precise identification of the most discriminating SNPs. As a result, the platform provides the minimal set of SNPs necessary for accession discrimination. For each identified SNP, users can access detailed information, including SNP ID, allele, and the number of its occurrences (Fig. 3). Additionally, users can view tables that demonstrate how different SNP selections progressively refine the list of candidate accessions. This functionality is especially valuable for developing targeted KASP assays, optimizing reagent usage, and minimizing the need for additional KASP assay mixes.

### Genotype string

This query mode allows users to compare a string of genotypes against those recorded in the database, enabling efficient identification and analysis of genetic similarities and differences. Input data must be provided as FASTA files, with SNP loci organized by chromosome and position. Each sequence string must include 38 symbols, with ‘N’ used to represent any unknown genotypes at specific loci. Once the data is submitted, the platform utilizes MAFFT to align the input sequences with the full set of genotypes in the database, ensuring precise positional matching of alleles across all genotypes.

**Figure 4.**
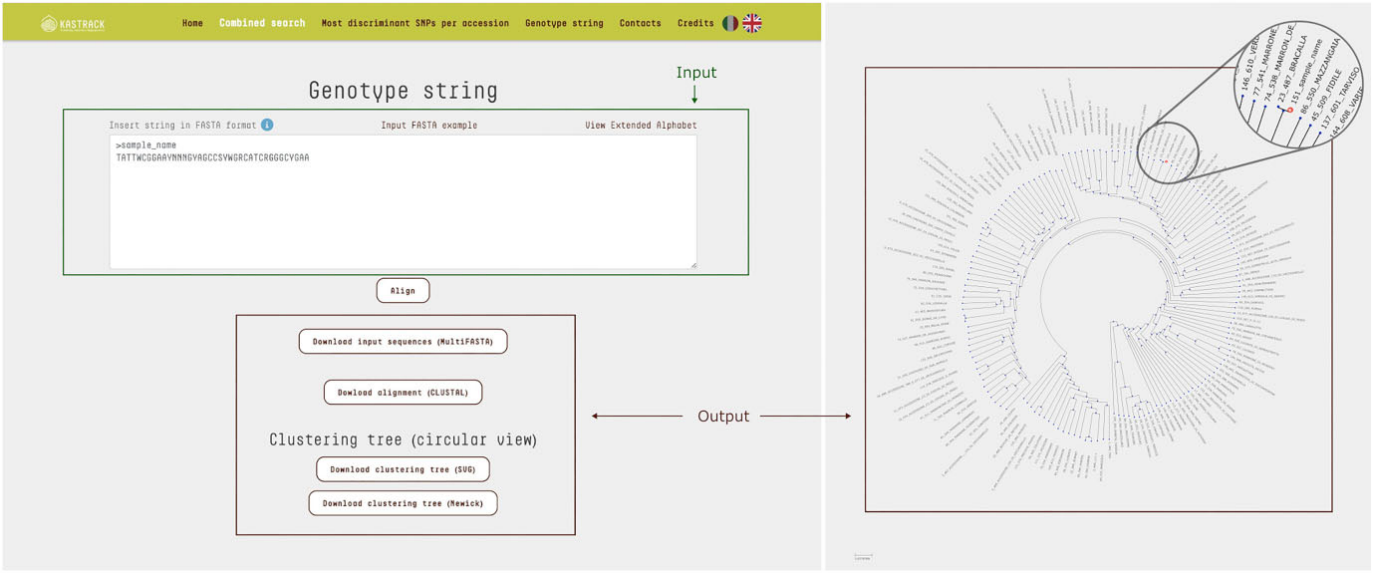
The ‘Genotype String’ query mode accepts a text input in FASTA format, consisting of SNP loci concatenated and ordered by chromosome and position. This input sequence is then compared against all entries in the database, generating a multiple sequence alignment in CLUSTAL format along with a corresponding cladogram tree. In the resulting tree, the branch representing the input sequence is highlighted different color, allowing for immediate visual identification. In the example shown, the input genotype string corresponds to a test sample that clusters closely with the accession ‘Bracalla’ in the cladogram.

The resulting clustering tree is displayed with the branch corresponding to the input sequence highlighted, allowing users to easily identify the accessions to which the input string is most similar (Fig. 4).

### SNP and accession pages

**Figure 5.**
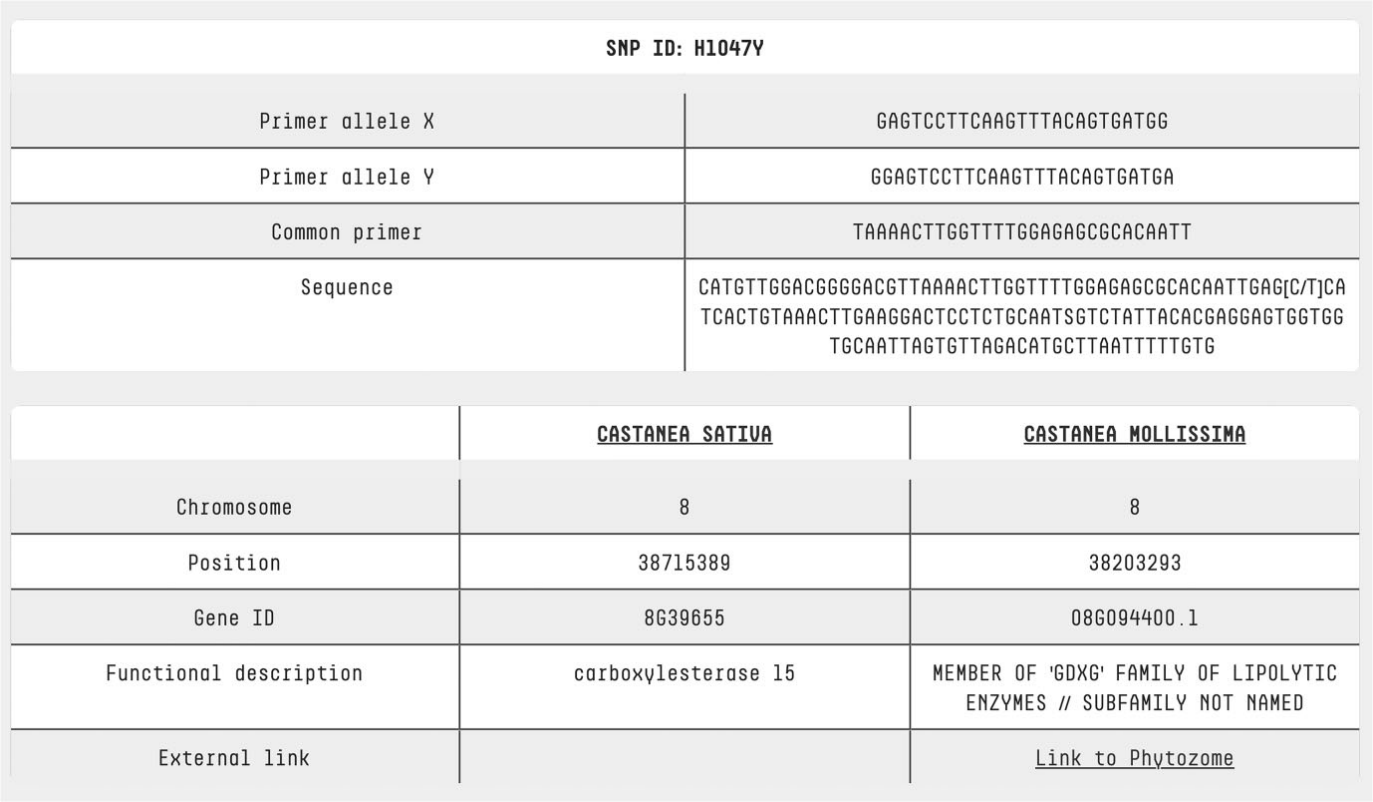
SNP page. At the top of the SNP page, detailed information is provided about the primers used for KASP analysis, along with the sequence containing the SNP of interest. This allows users to understand the assay design and sequence context. At the bottom of the page, annotation data from two reference genomes is presented, offering insights into each SNP’s genomic location, functional context, and potential biological significance. For Castanea mollissima, a direct link to a genome browser is also available, enabling users to explore the genomic context of each SNP in greater detail through an interactive visualization.

Users can access dedicated pages for SNPs and accessions by simply clicking on the SNP ID or accession name in the search result table (Fig. 2). The SNP page features a comprehensive table that includes: both forward primers, the common primer, the sequence containing the SNP, chromosome location, position, gene ID, functional description, and a cross-reference to the *C. mollissima* genome browser (Fig. 5). By clicking on an accession name, users are redirected to a detailed accession analysis page, which includes a brief description of the variety, its morphological traits, and photos representing the accession.

### KASTRACKdb usage example

KASTRACKdb provides comprehensive support for users across various scenarios encountered during sample analysis. The simplest case is when a sample is assumed to belong to a particular accession/cultivar. In this scenario, users can search for the most discriminating SNPs to gather the necessary information for developing a targeted KASP protocol. If the results of the analysis align with the data in the database, the sample can confidently be identified as belonging to the selected accession.

In cases where further analysis is needed, users can exploit the data obtained from the KASP analysis to perform discrimination through a combined search. By inputting known alleles and any observed morphological data, the platform will identify potential accessions to which the sample may belong. To refine the search and achieve a definitive identification, the software provides a list of SNPs with the highest PIC values, enabling users to focus on the most informative SNPs for targeted analysis.

A less common but important scenario occurs when the complete string of alleles does not match any cultivar in the database. In this case, users can input the string, containing concatenated SNP loci in FASTA format, into the ‘Genotype string’ search section. KASTRACKdb will then graphically visualize the similarity between the unidentified sample and the existing accessions in the database, allowing for further analysis and insights.

### Data download

Having data available for download is important for a database as it enables users to conduct further offline analysis, integrate findings into existing workflows, and ensure the reproducibility of their research. This accessibility maximizes the utility of the data across various applications. In KASTRACKdb, query results can be downloaded in text format (TXT), providing users with a simple and convenient way to manage, analyse, and integrate their data into various workflows. This feature is particularly valuable for developing targeted KASP assays. Additionally, the platform provides options to download alignments in Clustal format and clustering trees in multiple formats, including SVG and Newick, ensuring flexibility for diverse analytical needs.

## Discussion

For decades, the identification of chestnut varieties has relied on traditional morpho-physiological methods [25,26]. While these techniques have been foundational, they have significant limitations, such as their inability to accurately identify young, dormant, or scion trees and the need for specialized expertise, which is both costly to acquire and difficult to transfer [1,10]. In contrast, recent advancements in molecular tools, particularly SNP markers identified through KASP technology, have innovated cultivar identification, offering a more precise and efficient approach [12,21,22].

We have successfully developed KASTRACKdb, as an interactive genetic fingerprinting database that enhances chestnut cultivar/accession identification using KASP genotyping data.

To the best of our knowledge, no similar database is currently available, making KASTRACKdb a valuable resource for the entire scientific community working on chestnut. KASTRACKdb ensures accurate varietal identification, safeguarding intellectual property, verifying authenticity, and supporting commercialization. It also plays a key role in assessing genetic diversity, which is crucial for advancing breeding efforts and promoting the sustainable use and preservation of chestnut resources. This platform represents a significant step forward in the genetic characterization of chestnut and holds considerable potential for further expansion and integration with other resources. In future versions, we plan to expand the number of SNP loci to enhance the resolution of cultivar/accession identification, as well as increase the number of samples included in the database.

## Data Availability

The data underlying this article are available in KASTRACKdb at https://kastrack.crea.gov.it/kastrackdb/?lang=en.

## References

[1] Nunziata A, Ruggieri V, Petriccione M et al. Single nucleotide polymorphisms as practical molecular tools to support european chestnut agrobiodiversity management. Int J Mol Sci. 2020;21:1–19. 10.3390/ijms21134805 PMC7370276 32646057

[2] Ferrara E, Pecoraro MT, Cice D et al. A Joint approach of morphological and UHPLC-HRMS analyses to throw light on the autochthonous ‘Verdole’ chestnut for nutraceutical innovation of its waste. Molecules. 2022;27:8924. 10.3390/molecules27248924 36558057 PMC9785621

[3] Conedera M, Stanga P, Lischer C et al. Competition and dynamics in abandoned chestnut orchards in southern Switzerland. Ecol Mediterr. 2000;26:8924101–12.

[4] Pividori M, Armando F, Conedera M. Post-cultural stand dynamics in an abandoned chestnut coppice at its ecological border. In: Proceedings of the III International Chestnut Congress 693. p. 219–24. Bierbeek: International Society for Horticultural Science, 2004.

[5] Manetti MC, Becagli C, Carbone F et al. Linee guida per la selvicoltura dei cedui di castagno Rete Rurale Nazionale, Consiglio per la ricerca in agricoltura e l’analisi dell’economia agraria. Roma. 2017;3:275–95.

[6] Pereira-Lorenzo S, Ramos-Cabrer AM, Barreneche T et al. Instant domestication process of European chestnut cultivars. Ann Appl Biol. 2019;174:74–85. 10.1111/aab.12474

[7] Keys RN . Prospects for vegetative propagation in the genus *Castanea*. In: Proceedings of the American Chestnut Symposium. p. 10–16.Morgantown, WV: West Virginia University, 1978.

[8] Osterc G, Fras MZ, Vodenik T et al. The propagation of chestnut (*Castanea sativa* Mill.) nodal explants. Acta Agriculturae Slovenica. 2005;85:411–18. 10.14720/aas.2005.85.2.15252

[9] Martín MA, Mattioni C, Cherubini M et al. A comparative study of European chestnut varieties in relation to adaptive markers. Agrofor Syst. 2017;91:97–109.

[10] Calandrelli MM, Nunziata A, De Masi L. Pilot study on the geographical mapping of genetic diversity among European chestnut (*Castanea sativa* Mill.) cultivars in southern Italy. Plants. 2023;12:917. 10.3390/plants12040917 36840265 PMC9964929

[11] Mellano MG, Beccaro GL, Donno D et al. *Castanea* spp. biodiversity conservation: collection and characterization of the genetic diversity of an endangered species. Genet Resour Crop Evol. 2012;59:1727–41. 10.1007/s10722-012-9794-x

[12] Martín A, Alvarez JB, Mattioni C et al. On-farm conservation of sweet chestnut (*Castanea sativa* Mill.) in Andalusia. J Agric Sci Technol. 2011;5:33.

[13] Eriksson G, Pliura A, Villani F et al. Management of genetic resources of the multi-purpose tree species *Castanea sativa* Mill. Acta Hortic. 2005;693:373–86. 10.17660/ActaHortic.2005.693.47

[14] Martín MA, Mattioni C, Cherubini M et al. Genetic characterisation of traditional chestnut varieties in Italy using microsatellites (simple sequence repeats) markers. Ann Appl Biol. 2010;157:37–44.

[15] Galderisi U, Cipollaro M, Bernardo GD et al. Molecular typing of Italian sweet chestnut cultivars by random amplified polymorphic DNA analysis. J Hortic Sci Biotechnol. 1998;73:259–63. 10.1080/14620316.1998.11510973

[16] Pereira-Lorenzo S, Ramos-Cabrer AM, Barreneche T et al. Database of European chestnut cultivars and definition of a core collection using simple sequence repeats. Tree Genet Genomes. 2017;13:1–6.

[17] Bianco L, Fontana P, Marchesini A et al. The de novo, chromosome-level genome assembly of the sweet chestnut (*Castanea sativa* Mill.) Cv. Marrone Di Chiusa Pesio. BMC Genomic Data. 2024;25:64. 10.1186/s12863-024-01245-7 38909221 PMC11193896

[18] Xing Y, Liu Y, Zhang Q et al. Hybrid de novo genome assembly of Chinese chestnut (*Castanea mollissima*). GigaScience. 2019;8:giz112. 10.1093/gigascience/giz112 PMC6741814 31513707

[19] Wang J, Hong P, Qiao Q et al. Chromosome-level genome assembly provides new insights into Japanese chestnut (*Castanea crenata*) genomes. Front Plant Sci. 2022;13:1049253. 10.3389/fpls.2022.1049253 36518506 PMC9742463

[20] Hu G, Cheng L, Cheng Y et al. Pan-genome analysis of three main Chinese chestnut varieties. Front Plant Sci. 2022;13:916550. 10.3389/fpls.2022.916550 35958219 PMC9358723

[21] Dipta B, Sood S, Mangal V et al. KASP: a high-throughput genotyping system and its applications in major crop plants for biotic and abiotic stress tolerance. Mol Biol Rep. 2024;51:508. 10.1007/s11033-024-09455-z 38622474

[22] Rahman MZ, Hasan MT, Rahman J. Kompetitive allele-specific PCR (KASP): an efficient high-throughput genotyping platform and its applications in crop variety development. In: Molecular Marker Techniques: A Potential Approach of Crop Improvement. Singapore: Springer Nature, 2023, 25–54. 10.1007/978-981-99-1612-2

[23] Yang F, Lang T, Wu J et al. SNP loci identification and KASP marker development system for genetic diversity, population structure, and fingerprinting in sweetpotato (*Ipomoea batatas* L.). BMC Genomics. 2024;25:1245. 10.1186/s12864-024-11139-8 39719557 PMC11668102

[24] Nagy S, Poczai P, Cernák I et al. PICcalc: an online program to calculate polymorphic information content for molecular genetic studies. Biochem Genet. 2012;50:670–72. 10.1007/s10528-012-9509-1 22573137

[25] Furones-Pérez P, Fernández-López J. Morphological and phenological description of 38 sweet chestnut cultivars (*Castanea sativa* Miller) in a contemporary collection. Span J Agric Res. 2009;7:829–43. 10.5424/sjar/2009074-1097

[26] Dinis LT, Ferreira-Cardoso J, Peixoto F et al. Study of morphological and chemical diversity in chestnut trees (var. ‘judia’) as a function of temperature sum. CyTA J Food. 2011;9:192–99. 10.1080/19476337.2010.512394

